# Development and Validation of a Perceived Community Food Accessibility Measurement Questionnaire for Korean Older Adults

**DOI:** 10.3390/nu15194301

**Published:** 2023-10-09

**Authors:** Jisoo Hong, Narae Yang, Kirang Kim

**Affiliations:** Department of Food Science and Nutrition, College of Science and Technology, Dankook University, Cheonan 31116, Republic of Korea

**Keywords:** aged, food environments, fruit, vegetables

## Abstract

As the community food environment is known to be an important factor in healthy food choices, it needs to be measured, to increase awareness and improve healthy eating. The objective of this study was to develop a perceived community food accessibility measurement (P-CFAM) questionnaire applicable to older adults in Korea and evaluate its reliability and validity. The questionnaire was developed based on community food environment factors that were found to affect food choices in previous studies. We evaluated the internal consistency reliability, construct validity, and criterion-related validity. The subjects were 188 older adults for validity. The Cronbach’s alpha value for the reliability measure was 0.9. The confirmatory factor analysis to evaluate the construct validity of the questionnaire showed values close to those of the optimal model (GFI = 0.983, AGFI = 0.948, RMR = 0.004, NFI = 0.987, CFI = 0.996). Regarding the criterion-related validity, the P-CFAM questionnaire results were significantly related to objective measurement indicators such as the number of grocery stores, the travel time to a grocery store, and the intake of vegetables and fruit. In conclusion, the developed P-CFAM questionnaire was shown to be reliable and valid, and useful for assessing older adults’ perceptions of community food accessibility in Korea.

## 1. Introduction

In old age, individuals are more vulnerable to inadequate nutrition compared to in adulthood, due to physiological and digestive function decline, which can lead to a decreased food intake and related functions and an increased risk of developing chronic diseases and complications [1]. Maintaining a healthy diet is important for sustaining a healthy lifestyle, preventing disease, and recovering quickly after acute illness [1,2,3]. There are various factors that affect the dietary intake of older adults, including their personal health status, medication use, personal preferences, cultural norms, and social and environmental factors [1]. In particular, changes in the food environment affecting the risk of unhealthy eating behaviors and chronic diseases, due to rapid social and economic changes, have emerged as important factors to be studied [4].

The food environment refers to the physical, economic, policy, and socio-cultural factors that influence the choice of healthy food and affect nutritional status [5]. Various factors of the food environment, such as accessibility (the travel time and distance to grocery stores), availability (the adequacy of a healthy food supply, such as the number of stores selling healthy food near homes), and the diversity, quality, and price of food, can have positive or negative effects on food purchasing and usual intake patterns [6,7]. The ability to walk, use public transportation, or drive is associated with eating; however, as these abilities decline in older adults, the accessibility and availability of the local community food environment can become very important factors [8]. Previous studies reported that older adults’ food purchases were influenced by the type, number, and proximity of grocery stores, the price of the food sold at grocery stores, and the nutritional information environment [3,7,8,9,10]. Therefore, an understanding of the food environment in the local community would be helpful for older adults to lead a healthy life, and this provides a starting point for designing relevant food policies.

Previous studies have assessed the food environment within retail stores, tools for measuring food environment differences between regions, and the relationship between the food environment and dietary intake [11,12,13,14]. In South Korea, differences in eating behavior have been reported based on geographic accessibility [15]. While accessibility is a key factor in the food environment, a recent qualitative study found that, among Korean older adults, the factors in choosing a food store were small portion packaging, delivery services, and the availability of domestically produced products [16]. Therefore, it is limiting to evaluate the food environment based on accessibility alone. In addition, low-income individuals use cheaper grocery stores rather than the nearest grocery store [16]. A food environment measurement tool has been developed for older adults living in urban South Korea, which measured factors including how grocery stores operate, accessibility via public transportation, the availability of delivery and phone ordering, and the number of food items available [17]. However, this comprises many questions and is an objective indicator. In terms of measuring the food environment, objective measurement is widely used, but there is a need for tools that can better understand individual perceptions, because the concept of individual perception of the food environment is not well understood [11]. Recently, measurement tools for understanding individuals’ perceptions of the food environment have been studied, along with improvements in the food environment to maintain a healthy diet, in Japan and Spain [12,13]. In addition, even though various studies into developing tools to measure local food environments have been conducted, there is still a lack of reliable and valid measurement tools for local food environments in Korea, despite the increased interest in this area. Therefore, the objective of this study is to develop a community food environment measurement tool that is comprehensive, simple, and applicable to Korean older adults, based on the local food environment factors that significantly affect food choices that were identified in previous studies, and to evaluate its reliability and validity.

## 2. Methods

### 2.1. Study Population

We recruited 188 community-dwelling older adult volunteers as a convenience sample from a senior welfare center at an urban and gun (county) office in rural Korea. In our study, we categorized Seoul as an urban area and Cheongyang-gun as a rural area, in accordance with the guidelines outlined by the Local Autonomy Act. Ninety-six older adults were enrolled from the Mapo Senior Welfare Center in Seoul. The Mapo Senior Welfare Center provide meals, programs, and services such as a daycare service, education, etc., to prevent disease and promote health at no, or a low, cost. Participant recruitment was accomplished through bulletin board postings at the welfare center and outreach by welfare center staff. Ninety-two older adults were eligible to be participants in the Community-integrated Care Service in Cheongyang-gun. This is a service that provides meal delivery, transportation support, exercise support, and residential environment improvements to residents who need care, such as elderly people with chronic diseases or elderly people living alone. The study was reviewed and approved by the Institutional Review Boards of Dankook University. All participants provided written informed consent prior to participation in the study.

### 2.2. General Characteristics

The structured questionnaire collected information on sex, age, type of living arrangement (alone or with a partner), income level (less than KRW 500,000 per month, KRW 500,000–1,000,000 per month, or higher than KRW 1,000,000 per month), education level (no education, elementary school graduation, middle school graduation, or higher), current alcohol and smoking habits, recipient status of the Government Assistance Program (yes or no), disease status (the number of illnesses, which could include hypertension, diabetes, cancer, chronic lung disease, myocardial infarction, heart failure, angina pectoris, asthma, arthritis, stroke, or kidney disease), and food security status (food insecurity scores of 0–2, and food security scores of 3 or higher). The food security status was assessed using a self-reported food security questionnaire from the Korean National Health and Nutrition Examination Survey (KNHANES). 

### 2.3. Development of the P-CFAM Questionnaire

The development of the P-CFAM questionnaire was based on a previous literature review [3]. Caspi et al. [6] proposed five key food environment dimensions—availability, accessibility, affordability, accommodation, and acceptability—through a systematic review. These have been found to effectively reflect the perception of the food environment among rural older adults [3]. Based on the qualitative research conducted with Korean older adults, there were perceptual differences in some dimensions based on income levels, while the overall perception of the five dimensions of the food environment was similar. Additionally, the presence of “availability of domestically produced food” in the acceptability dimension and “availability of delivery services and small portion packaging sales” in the accommodation dimension emerged as significant factors [16]. In this study, we defined the five domains of the community food environment based on the meaningful response sentences and key words identified in the previous qualitative research [16]. The five domains are as follows: (1) availability, which refers to the sufficient availability of healthy food in local grocery stores; (2) accessibility, which examines the proximity of grocery stores to one’s home; (3) affordability, which assesses the appropriateness of food prices in local grocery stores; (4) accommodation, which evaluates whether local grocery stores meet the needs and satisfaction of the individuals; and (5) acceptability, which represents whether the food sold in local grocery stores meets the criteria for good quality. Each domain was assessed using a 4-point scale ranging from “strongly agree”, to “agree”, to “disagree”, to “strongly disagree”. Subsequently, the P-CFAM questionnaire domains were evaluated through focus group interviews with experts. 

### 2.4. Assessment of Dietary Intake

Participants were asked how frequently they ate fruit and vegetables to confirm the pattern of usual intake. There were nine frequency categories (ranging from “never or less than once a month” to “3 times or more a day”) in the food frequency questionnaire (FFQ). Each category was converted into a frequency of intake in times per day. For each food group, the criteria for a sufficient intake according to the frequency of consumption were as follows: fruit intake was defined as 2 or more servings per day for men and 1 or more servings per day for women; vegetable intake was defined as 8 or more servings per day for men and 6 or more servings per day for women; vegetable intake excluding kimchi and pickled vegetables was defined as 5 or more servings per day for men and 3 or more servings per day for women.

### 2.5. Reliability and Construct Validity Assessment

To assess the reliability, we analyzed the internal consistency using Cronbach’s alpha coefficient. To evaluate the construct validity of the five components (availability, accessibility, affordability, accommodation, and acceptability) of the P-CFAM questionnaire, a confirmatory factor analysis was conducted. Confirmatory factor analysis is a statistical analysis method used to verify whether a pre-specified factor structure based on existing theories or previous research results is appropriate for the other study population. The goodness of fit was evaluated using a structural equation model. This approach not only allows the testing of hypotheses regarding the factors on which the measured variables will load, based on existing theories and previous research, but also has the advantage of implementing the optimal model through statistical comparisons of various models that were suggested in previous studies [18]. To assess the goodness of fit of the model, Q (χ^2^/*df*, ≤3.0), GFI (goodness of fit index; ≥0.9), AGFI (adjusted GFI; ≥0.9), RMR (root-mean-square residual; ≤0.05), NFI (normed fit index; ≥0.9), and CFI (comparative fit index; ≥0.9) were used [19]. Q evaluates the overall model fit. GFI and AGFI calculate the proportion of variance explained by the estimated population covariance. RMR assesses the difference in the residuals between the sample covariance model and the hypothesized covariance model. NFI assesses the model by comparing the χ^2^ value between the model and the null model. An NFI value closer to 1 indicates a higher correlation between the variables. CFI is a modified form of NFI that can be applied when sample sizes are small [20].

### 2.6. Criterion-Related Validity Assessment

In the assessment of the criterion validity for the P-CFAM questionnaire regarding the availability and accessibility of food environment factors, the objective measurement indicators included the number of food stores (supermarkets, large supermarkets, convenience stores, and traditional markets) within a 250 m and 500 m radius of the participants’ residential areas, and the self-reported travel time to reach the food store that they typically use. For the number of food stores, we used Naver Map and counted the number of grocery stores within a 250 m and 500 m radius of each subject’s home address. For the self-reported travel time, survey participants were asked how long it took them to get to their most-frequented food store.

Kappa values were calculated to assess the level of agreement between the perceived availability and that based on the objective measurement indicators (the number of grocery stores). The overall criterion validity of the P-CFAM questionnaire was evaluated via analysis of the correlation of the criteria with the consumption frequency of fruit and vegetables, which have been reported in previous studies to be highly associated with the local community food environment [21]. 

### 2.7. Statistical Analysis

Categorical variables were expressed as frequencies and percentages, and were compared using Pearson’s chi-squared test or Fisher’s exact test, as appropriate. Continuous variables were expressed as mean and standard deviation, and were analyzed using the *t*-test or analysis of variance. The adequacy of fruit and vegetable intake was classified according to the consumption frequency recommended for older adults by the Korean Healthy Eating Index (KHEI) [22]. Logistic regression analysis was performed to determine whether the food environment can reflect the level of fruit and vegetable intake, showing odds ratios (ORs) and 95% confidence intervals (95% CIs). A multivariable model was adjusted for sex (male or female), age (65–74 years old, 75–84 years old, 85 years old or older), education level (no education, elementary school graduation, middle school graduation, or higher), income (less than KRW 500,000 per month, KRW 500,000–1,000,000 per month, or greater than KRW 1,000,000 per month), and food security (food insecurity scores of 0–2, and food security scores of 3 or higher). Confirmatory factor analysis was performed using AMOS 25.0 to evaluate the construct validity, and all other statistical analyses were conducted using IBM SPSS Statistics 26.0 for Windows (IBM Inc., New York, NY, USA). Statistical significance was tested at the level of *p* < 0.05.

## 3. Results

### 3.1. Characteristics of Subjects

The validity assessment was conducted with a sample of 188 participants. The characteristics of the participants by residential area are presented in Table 1. Rural residents were more likely to be older, be less educated, and have lower income levels, were more likely to have food security, and showed a low frequency of purchasing food. Rural residents had a lower intake of vegetables excluding kimchi and pickled vegetables and fruit. Rural residents were also likely to have an insufficient frequency of intake of vegetables excluding kimchi and pickled vegetables and fruit.

### 3.2. Development of the P-CFAM Questionnaire

The development of the P-CFAM questionnaire was based on previous qualitative research that evaluated the applicability of key food environment dimensions proposed, in a systematic review, for the older adult population. The P-CFAM questionnaire was validated through focus group interviews with experts. The specific questionnaire domains are presented in Table 2.

### 3.3. Reliability and Construct Validity Assessment of the P-CFAM Questionnaire

**Figure 1:**
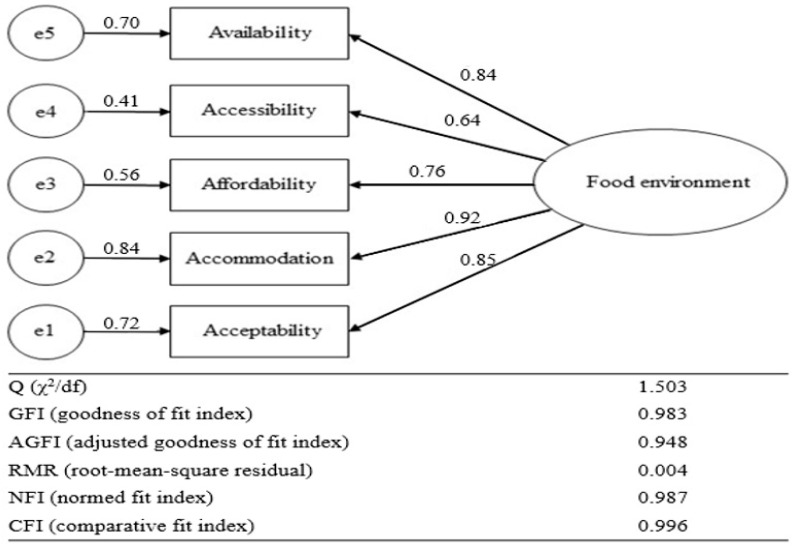
Results of the confirmatory factor analysis for content validity assessment.

In terms of internal consistency, the obtained Cronbach’s alpha value of 0.9 indicated high reliability. As a result of the confirmatory factor analysis, the factor loadings of domains were found to be between 0.64 and 0.92 (Figure 1). The fit indices for the model of the P-CFAM questionnaire were Q = 1.503, GFI = 0.983, CFI = 0.948, RMR = 0.004, NFI = 0.987, and CFI = 0.996. The values of all indices were close to those of the optimal model, indicating that the model succeeded in demonstrating goodness of fit to the data [3,23].

### 3.4. Distribution of the Perceived Community Food Accessibility Factors by Region

Table 3 shows the distribution of the perceived community food accessibility factors among urban and rural residents. Overall, the proportion of negative perceptions toward each food environment factor ranged from 51.4% to 72%, with the accessibility factor being perceived most negatively. The older adults in a rural setting perceived each community food environment factor more negatively than the older adults in an urban setting. Nearly half (47.6%) of the total participants and 87% of the rural residents responded negatively to all five factors of the food environment. 

### 3.5. Criterion-Related Validity Assessment of the P-CFAM Questionnaire

Table 4 shows the relationship between the perceived availability and accessibility factors in the P-CFAM questionnaire and the objective availability and accessibility indicators. There were regional differences in the relationship between the perceived availability and accessibility factors and the objective indicators. Of the study participants, 46.7% and 44% of older adults had no grocery stores within a radius of 250 m and 500 m from their homes, respectively, and all of them resided in rural areas. A higher number of grocery stores within a 250 m and 500 m radius was related to a more positive perception of availability factors. In rural areas in particular, the limited availability of grocery stores near people’s homes was significantly associated with a negative perception of availability factors. The lower quantity of grocery stores in rural areas and the negative perception of availability factors were significantly associated. The kappa values indicated a substantial agreement (0.557) between the availability factors and the objective indicators. Participants who perceived a negative level of accessibility had a significantly higher average travel time to their frequently visited grocery stores. In urban areas in particular, participants who perceived a negative accessibility had an average travel time of 23.4 min to grocery stores, which was significantly higher than the travel time of 7 min reported by participants who responded positively (*p* < 0.001).

### 3.6. Relationship between the Perceived Community Food Accessibility Factor and Inadequate Consumption of Vegetables and Fruit

Table 5 presents the multivariable-adjusted relationship between perceived community food accessibility and an inadequate consumption of vegetables and fruit. The odds of having an inadequate total vegetable intake, excluding kimchi and pickled vegetables, were higher in participants who negatively perceived the affordability, accommodation, and acceptability factors (OR = 2.48, 95% CI =1.16–5.30; OR = 3.56, 95% CI = 1.64–7.73; and OR = 2.31, 95% CI = 1.05–5.09, respectively). The odds of having an inadequate fruit intake were higher in participants who negatively perceived affordability and accommodation factors (OR = 3.09, 95% CI = 1.48–6.45, and OR = 2.33, 95% CI = 1.15–4.71, respectively). In rural settings, there was no relationship between the community food accessibility factors and the consumption of vegetables and fruit. In urban settings, the participants who negatively perceived the accommodation factor had higher odds of an inadequate total vegetable consumption, excluding kimchi and pickled vegetables (OR = 5.15, 95% CI = 1.34–19.81). In addition, the urban residents who negatively perceived the affordability factor had higher odds of an inadequate fruit consumption (OR = 3.84, 95% CI = 1.28–11.56). The participants who negatively perceived all the factors had greater odds of having an inadequate total vegetable intake excluding kimchi and pickled vegetables (OR = 3.04, 95% CI = 1.06–8.73). 

## 4. Discussion

As the food environment is reported to have a significant impact on people’s healthy food intake, it may be necessary to measure and monitor the food environment to assess levels of healthy food intake. We developed a perceived community food accessibility measurement (P-CFAM) questionnaire applicable to older adults in Korea. The developed P-CFAM questionnaire showed a high reliability and validity and was found to be useful in assessing the perception of community food accessibility among older adults in Korea.

Previous studies have developed and evaluated measurement tools for the food environment, which commonly address factors such as the availability, accessibility, acceptability, price, and quality of food, with a focus on grocery stores within the local community [11,12,13,14,17]. In particular, Caspi et al. proposed five key domains of the local community food environment, which were identified through systematic review, including availability, accessibility, affordability, accommodation, and acceptability [6]. Some studies have investigated whether the five domains of the food environment could be applied to older adults and have emphasized the need for such research [3,16,24].

The direct use in Korea of a measurement tool developed in foreign countries, without any consideration of cultural differences, may lead to potential misunderstandings, so it is necessary to evaluate the validity of the measurement questionnaire [25]. Therefore, we first confirmed the applicability of the five food environment domains proposed by Caspi et al. [6] (availability, accessibility, affordability, accommodation, and acceptability) to Korean older adults through qualitative research [16]. Then, in this study, a questionnaire was developed to ask about participants’ subjective perceptions, based on the five food environment domains that were previously identified to be applicable to older adults. Some studies have suggested that assessing the subjective perceptions of participants may be more meaningful and useful than assessing objective indicators [26,27]. The construct validity of a survey tool is commonly confirmed via exploratory factor analysis in the initial development stage to extract the key survey domains that reflect the concept of the target measurement. However, in this study, as the domains reflecting the concept of the measurement target were derived through theoretical and qualitative research, confirmatory factor analysis was conducted to verify the construct validity of the measurement questionnaire [18]. Confirmatory factor analysis can be considered a process of theoretical validation, as it places importance on the logical basis of previous studies and theoretical backgrounds [28,29]. In the evaluation of the overall goodness of fit of the model using the structural equation model in this study, all the goodness of fit indices indicated that the model met the criteria for a good fit.

This study found regional differences in food environmental factors. Among the food environmental factors, the proportion of subjects who perceived insufficient accessibility was high, especially among rural residents, who also had a higher negative perception of all food environmental factors. Previous Korean studies comparing the accessibility of food purchasing between urban and rural areas showed that urban areas have a higher variety of available groceries, making them more accessible, and have a greater number of large discount stores and supermarkets, indicating a better food environment than in rural areas [30]. Furthermore, people residing in the top 10% of areas with relatively good food accessibility were found to purchase groceries more frequently than those residing in the bottom 10% of areas with relatively poor food accessibility, which were predominantly rural areas [30]. This suggests that there may be potential effects of the disparity of food store environments between regions on the differences in health and nutritional status across regions [31].

Food environments are assessed through subjective measures of how participants perceive the food environment, and objective measures, such as the number and distance of food stores [23,32]. In this study, a higher proportion of participants in areas with fewer grocery stores had a negative perception of the availability factors of the food environment, and this was particularly the case among older adults in rural areas. Regarding the accessibility of grocery stores, participants with longer travel times to food stores had a higher negative perception of the accessibility factors of the food environment, which was significant among older adults in urban areas. Previous studies have reported differences between objective food environment indicators and subjective indicators [16,26,27]. In particular, urban areas, due to the development of public transportation, may include a wider range of accessible grocery stores for older adults, leading to greater differences in the perception of the food accessibility environment between public transportation users and non-users. On the other hand, in rural areas where there are commonly mobility constraints, there may be significant differences in the perception of the availability of nearby grocery stores, rather than the accessibility [33]. Therefore, considering that subjective perceptions of the food environment may vary depending on the local context, a comprehensive approach that includes both objective and subjective indicators could be more accurate in evaluating the community food environment [34].

Previous research has shown that food environments influence healthy food intake among older adults, particularly regarding patterns in their usual fruit and vegetable intake [7,35,36]. We collected dietary data using a FFQ to reflect the patterns in usual fruit and vegetable intake and classified those who consumed less than a cut-off frequency as an inadequate group. It has been found that older adults in rural areas are at higher risk of inadequate consumption of fruit and vegetables compared to those in urban areas, indicating significant disparities in consumption across regions [37,38,39]. In this study, in the comparison of the frequencies of vegetable and fruit consumption based on food environmental factors, significant differences were observed in terms of affordability, accommodation, and acceptability, with higher daily food intake frequencies among participants who had a positive perception of the food environment. Specifically, in the urban area, significant associations were found between vegetable intake excluding kimchi and pickled vegetables and accommodation factors, as well as between fresh fruit intake and affordability factors. However, no significant results were found in the rural area. The participants in the rural area showed limited variation in income levels, the number of grocery stores, and the types of grocery stores, which could have attenuated the effect of the food environment. Additionally, rural older adults may be self-sufficient in terms of food (including vegetables and fruit) from farming, which may result in a relatively less significant impact from the community food accessibility environment on food purchases and consumption compared to the case of urban older adults [40]. 

This study has limitations and raises the need for further research in developing a measurement tool to assess the food environment in local communities in Korea. Firstly, the study had limitations in terms of self-selection bias due to convenience sampling, the sample size, and the geographical coverage, which restrict the generalizability of the research findings. Secondly, the gender ratio of the survey participants is not exactly the same as the population structure in Korea, but the overall population of Korea has a lower proportion of elderly men than women (0.46% in the 70–79 age group and 0.37% in the 80–89 age group) [41], so our results can be interpreted as a partial reflection of the gender ratio in Korea. However, it is necessary to conduct future studies that consider the demographic structure. To apply the findings to local communities in general in Korea, further research on a broader range of food environments is needed. Despite these limitations, this study developed a measurement tool for assessing the food environment for Korean older adults based on a concept of community food accessibility that had previously been studied abroad, and validated its reliability and validity. 

## 5. Conclusions

This study found a relationship between older adults’ food environment and fruit and vegetable intake and suggested that the developed questionnaire has an appropriate reliability and validity for measuring the food environment of Korean older adults. It can serve as a tool for future research on the measurement of the food environment, focused on this population. It also suggests that this simple tool can be used to identify priority areas for policy interventions, to reduce the health disparities caused by differences in healthy food intake between regions, and to improve the food environment. Furthermore, to address ‘fresh and quality food deserts’, policies should consider the aspects of ‘affordability, accommodation, and acceptability’ rather than just ‘accessibility and availability’. This consideration may include efforts such as promoting local food and grocery stores and establishing public mobile vendors [42].

## Figures and Tables

**Table 1:** The general characteristics of the subjects for the validity assessment.

Variables	All	Urban	Rural	*p* ^(3)^
	188 (100) ^(1)^	96 (51.1)	92 (48.9)	
Gender				
Female	132 (70.2)	67 (69.8)	65 (70.7)	0.897
Male	56 (29.8)	29 (30.2)	27 (29.3)	
Age (years)				
65–74	55 (29.3)	44 (45.8)	11 (12.0)	<0.001
75–84	97 (51.6)	42 (43.8)	55 (59.8)	
≥85	36 (19.1)	10 (10.4)	26 (28.3)	
Type of living arrangement				
Alone	147 (78.2)	77 (80.2)	70 (76.1)	0.494
With a partner	41 (21.8)	19 (19.8)	22 (23.9)	
Household income				
≤KRW 500,000	108 (57.4)	30 (31.3)	78 (84.8)	<0.001
KRW 500,000–1,000,000	69 (36.7)	56 (58.3)	13 (14.1)	
≥KRW 1,000,000	11 (5.9)	10 (10.4)	1 (1.1)	
Education level				
No formal education	81 (43.3)	26 (27.1)	55 (60.4)	<0.001
Elementary school	58 (31.0)	31 (32.3)	27 (29.7)	
≥Middle school	48 (25.7)	39 (40.6)	9 (9.9)	
Food security				
Yes	141 (75.0)	60 (62.5)	81 (88.0)	<0.001
No	47 (25.0)	36 (37.5)	11 (12.0)	
Alcohol				
Yes	54 (28.7)	29 (30.2)	25 (27.2)	0.646
No	134 (71.3)	67 (69.8)	67 (72.8)	
Smoking				
Yes	16 (8.6)	10 (10.5)	6 (6.5)	0.328
No	171 (91.4)	85 (89.5)	86 (93.5)	
Government support program				
Yes	86 (47.0)	44 (46.3)	42 (47.7)	0.848
No	97 (53.0)	51 (53.7)	46 (52.3)	
Disease				
No	17 (9.0)	11 (11.5)	6 (6.5)	0.462
1	46 (24.5)	24 (25.0)	22 (23.9)	
≥2	125 (66.5)	61 (63.5)	64 (69.6)	
Food purchase frequency				
>1 times/week	29 (15.4)	26 (27.1)	3 (3.3)	<0.001
1 times/week	55 (29.3)	38 (39.6)	17 (18.5)	
Rarely	104 (55.3)	32 (33.3)	72 (78.3)	
Daily frequency of vegetables and fruit				
Vegetables	6.13 ± 4.53 ^(2)^	6.50 ± 3.41	5.73 ± 5.46	0.248
Vegetables excluding kimchi and pickled vegetables	3.38 ± 3.43	4.27 ± 3.01	2.46 ± 3.61	<0.001
Fruit	0.55 ± 0.64	0.68 ± 0.57	0.43 ± 0.69	0.008
Sufficiency of vegetable and fruit intake				
Vegetables				
Sufficient	76 (40.4)	42 (43.8)	34 (37.0)	0.343
Insufficient	112 (59.6)	54 (56.3)	58 (63.0)	
Vegetables excluding kimchi and pickled vegetables				
Sufficient	62 (33.0)	48 (50.0)	14 (15.2)	<0.001
Insufficient	126 (67.0)	48 (50.0)	78 (84.8)	
Fruit				
Sufficient	70 (37.2)	47 (49.0)	23 (25.0)	0.001
Insufficient	118 (62.8)	49 (51.0)	69 (75.0)	

^(1)^ N (%), ^(2)^ mean ± SD, ^(3)^ *p*-value for significant difference among urban and rural residents.

**Table 2:** Development of the P-CFAM questionnaire based on a previous literature review and studies of the content validity assessment.

Dimensions of Food Environment	Concept Based on the Systematic Review [6]	Description of the Food Environment Dimension in Older Adults [3]	Exploratory Study to Measure the Food Environment for Korean Urban Older Adults [16]	Development of a Questionnaire to Measure the Food Environment in This Study
Availability	“The adequacy of the supply of healthy food; examples in the food environment might include the presence of certain types of restaurants near people’s homes, or the number of places to buy produce”	“Available food sources are far away (Are there sources for food?)”	“Sufficiency of nearby grocery stores”	“The grocery stores in my neighborhood (near home) where I can buy a variety of healthy foods such as fruit and vegetables are sufficient”
Accessibility	“The location of the food supply and ease of getting to that location. Travel time and distance are key measures”	“Long distances, no transportation, compromised ability to get to food sources (Can individuals get to or make use of the food that is available?)”	“Utilization of grocery stores of 5–10 min walk” “Utilization of a traditional market near the subway”	“The grocery stores where I can buy a variety of healthy foods are close to my home”
Affordability	“Food prices and people’s perceptions of worth relative to the cost, often measured by store audits of specific foods, or regional price indices”	“Compromised ability to buy food due to income (Are individuals able to pay for the food that is available?)”	“Experience of restricted purchasing food from grocery stores near home because of food prices” “Differentiated strategies by household income level for economic and reasonable food purchase”	“The foods in my local (near home) grocery stores are affordable for me.”
Accommodation	“How well local food sources accept and adapt to local residents’ needs, for example store hours and types of payment accepted.”	“Vendors do not meet needs or recognize preferences (Do food sources respond to needs?)”	“Delivery service to home” “Small packages of food” “Promotion and provision of information about the product”	“The grocery stores in my neighborhood (near home) have good services (for example, small amount purchases, delivery availability, business hours, etc.) that meets my needs.”
Acceptability	“People’s attitudes about attributes of their local food environment and whether or not the given supply of products meets their personal standards”	“Budget forced to concede food preferences and available food fails to meet standards (Does the food available meet community standards?)”	“Freshness of foods” “Products of domestic origin” “A variety of food sale”	“The foods in my local (near home) grocery stores are fresh and of good quality.”

**Table 3:** Distribution of the perceived community food accessibility factors by region.

		All	Urban	Rural	*p* ^(2)^
Availability	Positive	76 (41.3) ^(1)^	64 (69.6)	12 (13.0)	<0.001
	Negative	108 (58.7)	28 (30.4)	80 (87.0)	
Accessibility	Positive	52 (28.0)	40 (42.6)	12 (13.0)	<0.001
	Negative	134 (72.0)	54 (57.4)	80 (87.0)	
Affordability	Positive	62 (34.3)	51 (57.3)	11 (12.0)	<0.001
	Negative	119 (65.7)	38 (42.7)	81 (88.0)	
Accommodation	Positive	81 (44.8)	69 (77.5)	12 (13.0)	<0.001
	Negative	100 (55.2)	20 (22.5)	80 (87.0)	
Acceptability	Positive	88 (48.6)	76 (85.4)	12 (13.0)	<0.001
	Negative	93 (51.4)	13 (14.6)	80 (87.0)	
Number of satisfied dimensions of the food environment	0	89 (47.6)	9 (9.5)	80 (87.0)	<0.001
	1	8 (4.3)	8 (8.4)	0 (0.0)	
	2	11 (5.9)	11 (11.6)	0 (0.0)	
	3	16 (8.6)	16 (16.8)	0 (0.0)	
	4	34 (18.2)	33 (34.7)	1 (1.1)	
	5	29 (15.5)	18 (18.9)	11 (12.0)	

^(1)^ N (%), ^(2)^ *p*-value for significant difference among urban and rural residents.

**Table 4:** Criterion-validity assessment of availability and accessibility among the perceived community food environment factors.

	All			Urban			Rural		
	All	Positive	Negative	All	Positive	Negative	All	Positive	Negative
Availability									
Number of grocery stores within a certain radius									
Within a 250 m radius									
0	85 (46.7) ^(1)^	7 (8.2)	78 (91.8)	0 (0.0)	0 (0.0)	0 (0.0)	85 (92.4)	7 (8.2)	78 (91.8)
1~50	85 (46.7)	61 (71.8)	24 (28.2)	78 (86.7)	56 (71.8)	22 (28.2)	7 (7.6)	5 (71.4)	2 (28.6)
>50	12 (6.6)	7 (58.3)	5 (41.7)	12 (13.3)	7 (58.3)	5 (41.7)	0 (0.0)	0 (0.0)	0 (0.0)
*p* ^(3)^		<0.001			0.335			<0.001	
*kappa value*		0.557			0.040			0.476	
Within a 500 m radius									
0	80 (44.0)	5 (6.7)	75 (70.1)	0 (0.0)	0 (0.0)	0 (0.0)	80 (87.0)	5 (41.7)	75 (93.8)
1~100	25 (13.7)	14 (18.7)	11 (10.3)	13 (14.4)	7 (11.1)	6 (22.2)	12 (13.0)	7 (58.3)	5 (6.3)
>100	77 (42.3)	56 (74.7)	21 (19.6)	77 (85.6)	56 (88.9)	21 (77.8)	0 (0.0)	0 (0.0)	0 (0.0)
*p* ^(3)^		<0.001			0.198			<0.001	
*kappa value*		0.254			−0.026			0.521	
**Accessibility**									
Self-reported travel time to a grocery store (min)	16.52 ± 15.40 ^(2)^	7.88 ± 5.02	20.50 ± 16.89	16.42 ± 14.57	6.99 ± 4.61	23.41 ± 15.51	16.66 ± 16.58	11.05 ± 5.33	17.74 ± 17.79
*p* ^(3)^		<0.001			<0.001			0.223	

^(1)^ N (%), ^(2)^ mean ± SD, ^(3)^ *p*-value for significant difference among urban and rural residents.

**Table 5:** Relationship between the perceived community food environment factors and an inadequate vegetable and fruit intake frequency for criterion-validity assessment ^(1)^.

		Vegetables			Total Vegetables Except Kimchi and Pickled			Fruit		
		Total	Urban	Rural	Total	Urban	Rural	Total	Urban	Rural
Availability	Positive	1.00	1.00	1.00	1.00	1.00	1.00	1.00	1.00	1.00
	Negative	1.27 (0.65–2.49) ^(2)^	1.06 (0.38–3.00)	3.09 (0.70–13.62)	1.65 (0.80–3.40)	0.84 (0.30–2.40)	2.83 (0.45–17.66)	1.36 (0.69–2.70)	0.95 (0.34–2.64)	0.70 (0.14–3.54)
Accessibility	Positive	1.00	1.00	1.00	1.00	1.00	1.00	1.00	1.00	1.00
	Negative	1.06 (0.51–2.2)	0.83 (0.31–2.21)	3.09 (0.70–13.62)	1.02 (0.47–2.22)	0.73 (0.28–1.92)	2.83 (0.45–17.66)	1.30 (0.63–2.71)	1.34 (0.52–3.49)	0.70 (0.14–3.54)
Affordability	Positive	1.00	1.00	1.00	1.00	1.00	1.00	1.00	1.00	1.00
	Negative	1.67 (0.83–3.36)	1.89 (0.65–5.44)	2.59 (0.56–12.02)	2.48 (1.16–5.30)	2.00 (0.67–5.95)	4.62 (0.64–33.54)	3.09 (1.48–6.45)	3.84 (1.28–11.56)	0.84 (0.16–4.42)
Accommodation	Positive	1.00	1.00	1.00	1.00	1.00	1.00	1.00	1.00	1.00
	Negative	1.91 (0.95–3.81)	3.15 (0.87–11.39)	3.09 (0.70–13.62)	3.56 (1.64–7.73)	5.15 (1.34–19.81)	2.83 (0.45–17.66)	2.33 (1.15–4.71)	3.17 (0.95–10.55)	0.70 (0.14–3.54)
Acceptability	Positive	1.00	1.00	1.00	1.00	1.00	1.00	1.00	1.00	1.00
	Negative	1.37 (0.67–2.78)	0.96 (0.24–3.76)	3.09 (0.70–13.62)	2.31 (1.05–5.09)	1.40 (0.34–5.79)	2.83 (0.45–17.66)	1.51 (0.73–3.14)	0.92 (0.22–3.82)	0.70 (0.14–3.54)
The number of negatively perceived dimensions	0	1.00	1.00	1.00	1.00	1.00	1.00	1.00	1.00	1.00
	1–4	1.03 (0.40–2.65)	1.32 (0.41–4.29)	-	0.98 (0.37–2.59)	1.34 (0.41–4.35)	-	0.86 (0.33–2.22)	0.98 (0.31–3.08)	-
	5	1.57 (0.60–4.05)	3.01 (0.11–83.75)	2.67 (0.58–12.30)	3.04 (1.06–8.73)	3.50 (0.13–92.11)	4.53 (0.62–33.25)	1.68 (0.63–4.52)	2.55 (0.14–45.97)	0.82 (0.16–4.32)

^(1)^ Adjusted for sex, age, education level, household income, and food security. ^(2)^ The odds ratio and 95% confidence interval were obtained via logistic regression analysis.

## Data Availability

Not applicable.
